# Improvement in Durability and Mechanical Performance of Concrete Exposed to Aggressive Environments by Using Polymer

**DOI:** 10.3390/ma15113751

**Published:** 2022-05-24

**Authors:** Maria Idrees, Arslan Akbar, Farhan Saeed, Huma Saleem, Tousif Hussian, Nikolai Ivanovich Vatin

**Affiliations:** 1 Department of Architectural Engineering & Design, Faculty of Civil Engineering, University of Engineering and Technology, Lahore 54890, Pakistan; saleemh40@gmail.com; 2 Department of Architecture and Civil Engineering, City University of Hong Kong, Kowloon, Hong Kong 999077, China; 3 Department of Polymer Engineering, University of Engineering and Technology, Lahore 54890, Pakistan; f.saeed@uet.edu.pk; 4 Centre for Advanced Studies in Physics, Government College University, Lahore 54890, Pakistan; tousifhussain@gcu.edu.pk; 5 Peter the Great St. Petersburg Polytechnic University, 195291 St. Petersburg, Russia; vatin@mail.ru

**Keywords:** styrene-butadiene-rubber, cement-based materials, durability, sustainability

## Abstract

Concrete is the most widely used construction material. However, it cannot sustain the harsh environment and can easily deteriorate. It results in repair and reworks that amount to a considerable loss of money and time. The life span of concrete reduces if exposed to external attacks, for instance, sulfate attacks, alkali-silica reactions, corrosion, and drying shrinkage. These ubiquitous attacks cause a reduction in service life and raise the need for early repair and maintenance, resulting in higher life cycle costs and structural failures. To resolve these issues, the potential of styrene-butadiene-rubber (SBR) ultrafine powder as cement replacement polymeric admixture at 0%, 3%, 5%, 7%, and 10% have been evaluated. The effect of SBR-powder on concrete is investigated by conducting an alkali-silica reactivity test (ASR), rapid-chloride-permeability test (RCPT), drying shrinkage, and sulfate resistivity tests. Workability, compressive and flexural strength tests are also conducted. For ASR and drying shrinkage, mortar bar samples were cast, exposed to respective environments, and the percentage change in length was measured. For mechanical tests and RCPT, prisms, cylinders and cubes were cast and tested at 28 days. The SBR-powder modification reduces concrete’s permeability, drying shrinkage, and expansions due to ASR and sulfate attacks. SBR powder increased workability by 90%, compressive strength by 23%, and flexural strength by 9.4% in concrete when used at 10% cement replacement by weight. The SBR-powder (10%) modification reduced the RCPT value by up to one-third (67%), drying shrinkage by 53%, ASR by 57%, and sulfate reaction by 73%. Consequently, SBR powder usage can adequately improve the workability, mechanical properties, and durability of the concrete and lead to advanced sustainable concrete with low repair requirements.

## 1. Introduction

Concrete is the most widely used construction material, generally composed of cement, crush, and sand. Its benefits and usability usually overshadow its negative impact on the environment. Environmental scientists are continuously struggling to abate the negative impact of cement and concrete production. This research explores expensive carbon capture technologies/sequestration and injecting liquid carbon dioxide to achieve a low carbon footprint.

Sustainable and durable concrete for long-term performance and improved mechanical properties is a dire need of the present and future [1,2]. Producing long-life concrete is one of the most pragmatic ways to reduce the carbon footprint and conserve aggregate resources. For instance, if concrete life is doubled, then the demand for aggregates and cement reduces to half. Long-life durable concrete also reduces the duration and resources spent on repairs and construction. It also reduces the demand for cement. Thus, it will mitigate CO_2_ emissions and reduce energy consumption, leading to high sustainability and energy efficiency.

The durability of concrete allows it to resist weathering action, chemical attacks, and physical deterioration without compromising its engineering properties. The durability and longer life span of concrete depend mainly on permeability, i.e., the interconnectivity of concrete pores [3,4,5]. Its permeability and cracking need to be controlled to achieve higher service life and the sustainability of concrete at low maintenance costs [6,7].

Durability and service life design have become key factors, especially in extreme environments [8,9]. Concrete may be easily affected by the harsh environment and deteriorate quickly. Concrete laid in areas affected by sulfate attacks may not deteriorate quickly. Similarly, concrete laid using a reactive aggregate may deteriorate easily. The life span of concrete can be reduced from 100 years to hardly ten years or less due to deterioration and other chemical attacks. It may lead to rapid repairs and reconstructions, thus increasing the utilization of concrete ingredients further many folds. These external attacks and durability issues reduce the service life and cause loss of money and time and the need for repair and maintenance.

Admixtures (such as polymers) that can reduce permeability should be explored in extreme environments to resolve durability issues [10] and increase the life of structures. Epoxy resin (liquid)-modified concrete and polymer concrete (without cement) provide better mechanical properties and durability. However, their use is limited due to the high cost [11,12]. SAP (super absorbent polymers) increases durability but reduces compressive strength [13,14]. Polymer-incorporated concrete shows exceptionally good performance in terms of durability when exposed to harsh environments [15]. Polymers are used in repair mortars due to their intrinsic properties [16].

Ultrafine SBR powder is used in the study because it can prove beneficial in reducing permeability. The properties of SBR depend on the styrene and butadiene ratio [17]. It has better durability, decreased shrinkage, better bond strength, increased flexibility, and better water resistance and chloride ion penetration resistance [6,18,19]. Additionally, SBR has a lower degradation rate than cement mortar [20]. With the increase in SBR quantity, the flexural strength increases gradually due to the better bond of the interfacial transition zone (ITZ) and aggregate in the presence of SBR. Moreover, SBR can effectively be used in 3D printing concrete using a higher SBR/cement ratio to obtain desired properties [21,22].

The presented research aims to evaluate the performance of concrete against environmental issues such as alkali-silica reactivity, drying shrinkage, and sulfate resistivity to ensure long life. Ultrafine SBR powder at 0%, 3%, 5%, 7%, and 10% cement replacement is used. The slump value and compressive and flexural strengths are also determined. SBR powder was expected to improve the durability and physical properties of concrete and mortar. This study implies the solution to the early deterioration of concrete and environmental issues. The production of concrete with low repair and reconstruction demand leads to reduced cement usage, lower carbon footprint, better resources conservation, and higher energy efficiency.

The study devises a modified concrete composition, which will not be significantly affected in harsh/ aggressive environments. Due to its intrinsic properties, the selected polymer compensates for the deficiencies in concrete. Thus, the modified concrete can withstand environmental conditions well without repeated repair and reworks and loss of time and money. Otherwise, repairing or replacing existing components due to exposure to an aggressive environment is very expensive and technically difficult [23].

## 2. Materials and Methods

### 2.1. Materials

Ordinary Portland cement Type-1 conforming to ASTM C-150 with a 3078 cm^2^/g blain fineness was used as a hydraulic binder to produce mortar and concrete samples. Table 1 shows the physical and chemical properties of hydraulic cement and the properties of coarseness and fine aggregate. The physical properties were obtained by conducting tests, while the chemical composition was found by XRF analysis.

The properties of ultrafine styrene-butadiene-rubber (SBR) powder are shown in Table 2. Ultrafine SBR re-dispersible powder used in this study is of size 85 micron (one-tenth of ordinary SBR powder). Re-dispersible powders are formed by spraying and chemically treating the liquid latex [24]. The particle size of commercially available solid particles in liquid latex polymer is usually 0.15 microns. Hence, the effect of SBR is dissimilar to the properties of concrete depending on solid particle sizes. Idrees et al. described the phenomenon of increased compressive strength of SBR-powder-modified mortar, contrary to the decreased compressive strength of SBR-latex-modified mortar after 5% of cement replacement [25]. Moreover, SBR powder can also be used as an additive in producing pre-packaged cement mixes and preparing specific performance ready-made concrete mixes.

### 2.2. Methods

#### 2.2.1. Mix Proportion

The mix proportions of concrete for compressive strength, flexural strength, and rapid chloride permeability test samples are given in Table 3. Cement is replaced by styrene-butadiene-rubber (SBR) by up to 10% by weight because this was found to be an optimum value for replacement [25]. Higher loading of SBR may not be economical and feasible, so SBR is limited to 10% replacement considering the economy, feasibility, and previous work. Separate mixes were prepared for durability tests according to their relevant standards. Their composition is described in the following sections.

The mix proportions were selected for all durability tests, as described by corresponding relevant ASTM standards.

#### 2.2.2. Preparation of Samples

The concrete samples prepared were 4″ × 4″ × 20″ (100 mm × 100 mm × 10 mm) prisms for flexural strength testing, 2.75″ × 2.75″ × 2.75″ (70 mm × 70 mm × 70 mm) cubes for compressive strength tests, and 4″ × 2″ (100 mm × 50 mm) cylinders for rapid chloride permeability test. The mortar bars for sulfate resistivity, alkali-silica reactivity, and drying shrinkage were 1″ × 1″ × 11¼″ (25 mm × 25 mm × 285 mm) in size. Mortar cubes of size 2″ × 2″ × 2″ (50 mm × 50 mm × 50 mm) were also prepared for compressive strength tests related to sulfate attacks and for water absorption. The compressive strength and flexural strength tests were conducted in accordance with ASTMC109 and ASTMC78. Mechanical strength tests and RCPT tests were conducted after curing samples for 28 days [26,27]. However, for alkali-silica activity, the mortar bars were dipped into NaOH solution for 14 days at 80 °C. For sulfate resistivity, the samples were cured until they had a 20 Mpa strength. Then, they were dipped in sulfate solution for the remaining time.

**Figure 1:**
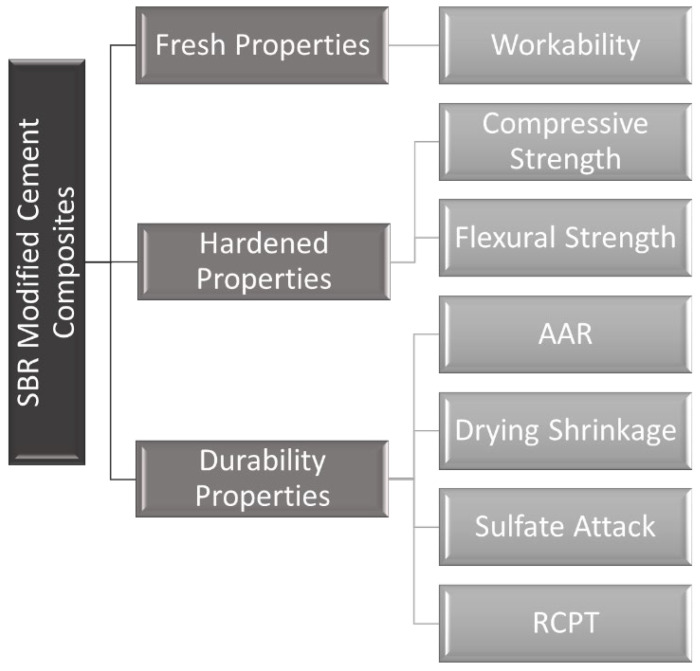
List of properties of SBR modified cement composites studied in this experimental program.

Figure 1 presents the experimentation conducted. Styrene-butadiene-rubber (SBR) powder percentages at 0%, 3%, 5%, 7%, and 10% with respect to cement weight were used to partially replace cement. During experimentation, the average laboratory temperature and relative humidity values were 33 °C (91.4 °F) and 57%, respectively. In the fresh state, slump tests were performed for SBR modified concrete samples. In the hardened state, flexural and compressive strength, rapid chloride permeability, alkali-silica reactivity, drying shrinkage, and sulfate resistivity tests for different concrete compositions were performed.

#### 2.2.3. Workability

A slump test was performed to assess the workability of concrete in conformance to the ASTMC143-78 standard. A conical steel mold was used to measure the slump value of concrete [28]. The concrete constituents cement, sand, and aggregate were taken in a ratio of 1:1.7:2.5, while the water–cement ratio was taken as 0.47.

#### 2.2.4. Mechanical Strength

Flexural strength test:

Higher flexural strength implies better cracks resistance, and it is directly related to the initiation of cracks. Flexural strength tests were carried out on concrete prisms conforming to ASTM C78 [27]. Four prisms for each composition were cast and tested after curing for 28 days.

Compressive strength test:

Compressive strength tests on four concrete samples for each mix composition were performed with the help of a universal testing machine. Four samples were cast and tested for compressive strength after curing for 28 days. The compressive strength test was conducted in accordance with ASTMC109 [26].

#### 2.2.5. Durability Issues and External Attacks

**Rapid chloride permeability test (RCPT) and Water absorption test:**

Permeability is the interconnectivity of concrete pores, which allows the water and other reagents to ingress concrete. The ingression of water and external reagents/ions in concrete causes various reactions and expansions that generate tensile stresses. As concrete has a low tensile strength, it cracks. Cracks, in turn, allow further ingression of ions and lead to higher deterioration [29]. Thus, decreased permeability and increased tensile strength are the keys to highly durable concrete and a longer life span.

Permeability is a crucial factor for durability in a corrosive environment [30]. RCPT test is a good indicator of permeability. Chloride ions from NaCl solution are forced to pass through a concrete cylinder (50 mm × 100 mm) toward the NaOH solution by applying a voltage difference of 60 V across two faces of a cylinder. The amount of charge passed in 6 h indicates the chloride ion permeability of the concrete. The total charge passed in coulombs was determined and compared for SBR-powder-modified samples in this investigation. An RCPT test was conducted conforming to ASTMC1202 [31]. A simple water absorption test on mortar samples (50 mm × 50 mm × 50 mm) was also conducted. Despite the advanced RCPT test, a simple water absorption test was conducted to have an idea of how much water is absorbed in samples in twenty-four hours. Thus, water absorption capacity was calculated by oven drying the sample and then dipping the same sample for 24 h. In this way, the percentage of water absorbed by the oven-dried sample within 24 h was calculated. The percentage increase in weight of the sample was its water absorption capacity.

**Alkali-silica reactivity test:**

This test method provides a means of detecting the potential of alkali-silica reactions that causes potentially deleterious internal expansion. The favorable condition for alkali-silica reactions was provided by crushing with reactive silica while preparing the bar samples. The bars were dipped in alkali solution for 14 days at an elevated temperature of 80 °C. The high temperature provided accelerated alkali-silica reaction. The test determined how this alkali-silica reactivity was affected by SBR powder (shown in Table 4). The test was conducted conforming to ASTMC1260 and C490 standards [32,33]. Mortar constituents’ cement and sand were taken in a ratio 1:2.25, while the water/cement ratio was taken as 0.47, conforming to standards.

**Drying shrinkage test:**

The drying shrinkage is affected by temperature, relative humidity, and rate of evaporation. This test method determines the shrinkage/reduction in length of mortar bars that are demolded after 24 h, kept for the next 48 h in a lime water bath, dried, and air stored. The test was conducted in hot, dry weather at 33 °C (91.4 °F) and 57% Relative Humidity (room atmosphere in June). The change in the lengths of mortar bars was measured at 7, 14, 21, and 28 days of their production (see Table 5). The testing was conducted conforming to ASTM C596 [34]. Mortar constituent’s cement and sand were taken in a ratio 1:2.25, while the water/cement ratio was taken as 0.47, conforming to standards.

**Sulfate resistivity test:**

This test method determines the expansion in length of mortar bars due to sulfate attacks. For sulfate resistivity test, mortar sample of mix ratio as given in Table 6 were prepared. Mortar bars were cured until the mortar cubes from the same batches attained a compressive strength of 20.0 MPa (3000 psi). Then, the bars were dipped in sulfate solution, and the expansion in length of bars was measured regularly for fifteen weeks. The test was conducted conforming to ASTM C1012 and C490 [33,35]. The mortar constituent’s cement and sand were taken in a ratio 1:2.75, while the water/cement ratio was taken as 0.485, conforming to standards.

#### 2.2.6. Scanning Electron Microscopic (SEM) Studies

SEM was used to study the microstructure of modified concrete on the 28th day of casting. The microstructure of the sample was analyzed by SEM-JEOL-JSM-4680LV at a working voltage of 15 KV. Sample was mounted on a stub of aluminum with carbon tape and was carbon-coated. The sample was carefully examined.

## 3. Results and Discussions

The experimental results are shown in the section below.

### 3.1. Workability of Concrete

**Figure 2:**
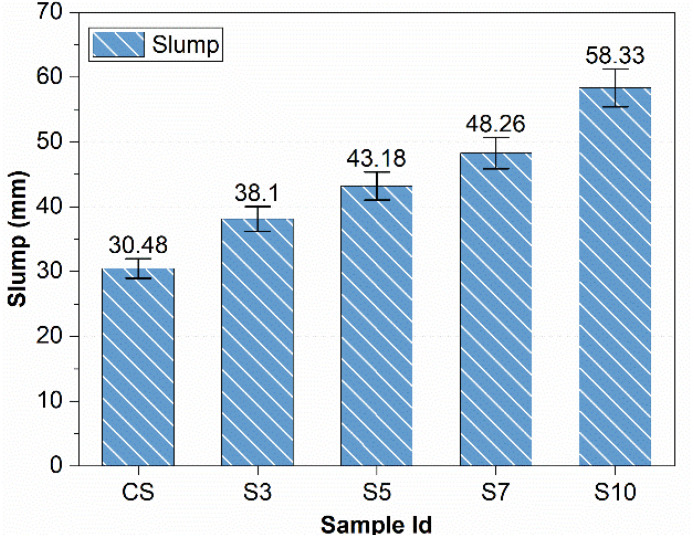
Concrete Slump Values (ASTM C143/C143M).

The slump value of concrete increased with the increase in styrene-butadiene-rubber (SBR) percentage, as shown in Figure 2. The modified concrete sample at 10% SBR powder showed a 90% higher slump than the control sample. SBR latex increases workability by increasing consistency due to polymer microstructure and reducing the drying of the mix [36]. The surfactants present in the polymer may act as plasticizers to increase the slump value and thus reduce the amount of water required. An increase in workability may also be associated with the polymer chains of SBR that facilitate the relative movement between the cement and other particles [37]. The ball-bearing effect of SBR powder round particles also contributes to increasing workability [10,38].

### 3.2. Mechanical Properties

**Flexural Strength:**

**Figure 3:**
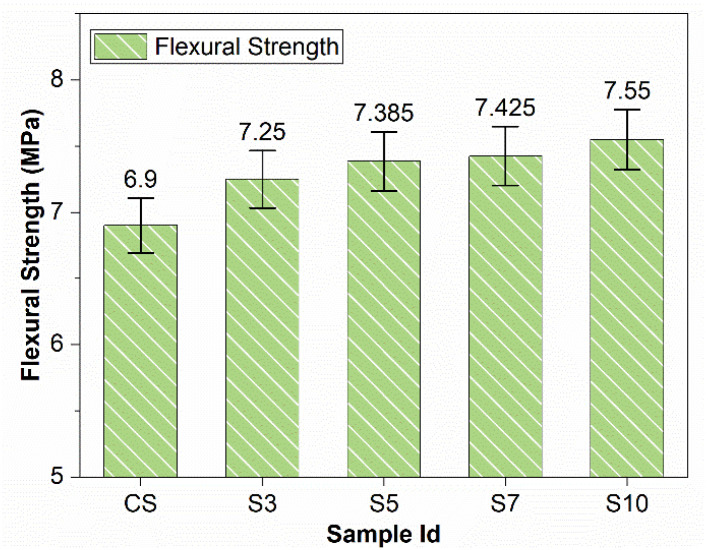
Flexural Strengths of Modified Concrete (ASTM C78).

Increasing the SBR powder percentage increased the values of flexural strengths, as shown in Figure 3. The maximum value of percentage increase for flexural strength, i.e., 9.4%, was obtained for 10% SBR powder. SBR powder might have improved the internal structure of concrete. In addition, the improvement in the transition zone as a result of the adhesion of the SBR powder might have increased tensile and flexural strength, along with good ductile behavior [39]. Similar results were obtained by Bhogayata et al., claiming that a higher SBR/cement ratio shows a higher flexural strength [40]. Higher flexural strength is the intrinsic quality of rubbers. This increase in flexural strength may be due to better bonding and the intrinsic nature of SBR polymer (i.e., rubber in nature).

**Compressive strength:**

**Figure 4:**
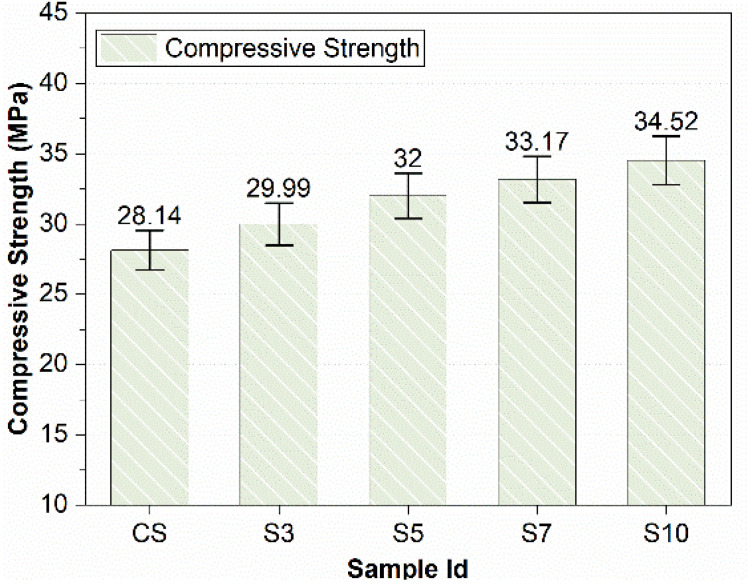
Compressive Strength of Modified Concrete.

In concrete samples with 0%, 3%, 5%, 7%, and 10% SBR modification, the compressive strength increased gradually with a relative increase in the percentage of SBR powder. Figure 4 shows that the compressive strengths of concrete increased with an increase in SBR percentage. Similar results were obtained for mortar in a recent study by the authors [25].

The maximum value of percentage increase in compressive strength was 23%, as shown in Figure 4. The SBR powder decreases the porosity and pore size because of the void-filling effect. In addition, SBR powder has anadhesive quality. It might act as an adhesive when mixed with water and might block the pores. Consequently, the compressive strength might be improved. A scanning electron microscopic (SEM) image in the following sections bolsters the result by showing very dense microstructure and hard SBR agglomerates.

A previous detailed study conducted by the author revealed that the compressive strength of mortar with SBR liquid latex reduces with an increase in SBR percentage after 5% cement replacement. The reason was the thick layer of polymer stopping further hydration and easy slip-ability between layers of polymers. This study is about SBR powder instead of SBR latex; the compressive strength does not decrease and keeps on increasing with the increasing percentage of SBR powder. The compressive strength kept increasing but is optimum for 5 to 10% utilization.

### 3.3. Permeability and Durability

**Figure 5:**
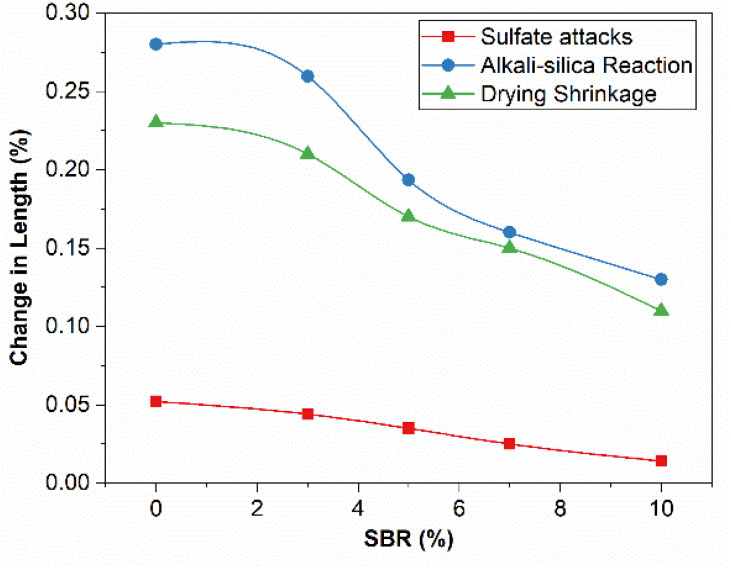
Variation in Length (%) of Mortar Bars Due to Expansions Caused by Sulfate Attacks and ASR, and Shrinkage Caused by Drying (ASTMC 490).

Sulfate attacks and alkali-silica reactions both cause expansion and thus cracking in concrete [41,42]. In contrast, drying shrinkage caused by loss of capillary water produces cracks due to shrinkage [43]. Standard test procedures are adopted for this purpose. The expansion and shrinkage in mortar bars exposed to harsh environments are studied. Figure 5 shows the change in length of 1″ × 1″ × 11¼″ mortar bar caused by the expansions due to sulfate attacks, alkali silica reactions, and shrinkage due to drying at ambient temperature. The change in length expresses volume instability due to attacks. The lower the change in length, the better the mortar/concrete against harsh environments.

**Rapid chloride permeability test (RCPT):**

**Figure 6:**
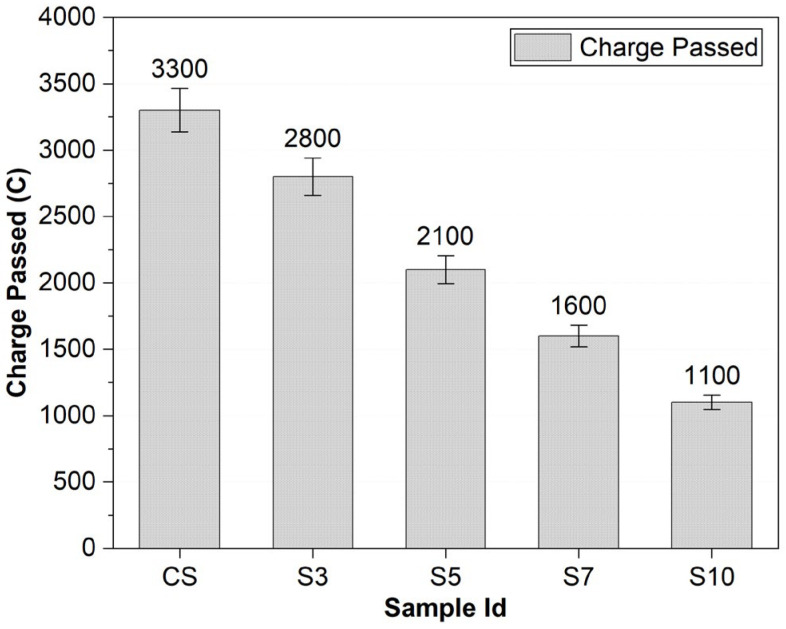
Average Charge Comparison of RCPT Specimens (ASTM C1202).

Figure 6 depicts the comparison of average charges passed through samples. Concrete samples with higher SBR powder content showed a lower amount of charge passing through them, while 10% SBR-modified concrete showed an exceptionally lower value of charge passed through it. The lower ingress of chloride ions depicts lower permeability and increased durability in concrete due to the reduction in the interconnectivity of concrete pores. The polymer fills pores and reduces permeability considerably, resulting in the reduction in chloride ion ingress. Hence, the lesser charge passes through SBR modified concrete samples. Lower chloride permeability implies a lower chance of corrosion caused especially by chloride ion ingress near the coastal area. At 10% replacement, the rapid chloride permeability was reduced by 67%. The results are similar to Bhogayata (2018) and Moodi’s (2018) findings that SBR-latex provides excellent resistance against chloride ingression [40,44].

**Alkali-silica reactivity test (ASR):**

Table 7 and Figure 5 show the expansions of SBR-modified mortar bars caused by alkali-silica reactions.

The most favorable environment for ASR reactions was provided using NaOH alkali solution and aggregates containing reactive silica naturally. Hence, even a slight reduction in mortar bar expansion caused by ASR means that admixture provides better resistance against alkali-silica-reactivity. When alkali from the surrounding, e.g., cement, comes in contact with an aggregate with reactive silica, it forms an alkali-silica gel. So, expansion takes place. Due to the lower tensile strength of concrete, this expansion causes cracking. The mortar bars with 5% and higher cement replacement percentages with SBR powder showed the expansion within the limiting range, i.e., less than 0.2% expansion at 28 days (as suggested by ASTM standard). Overall, 5% of SBR reduced ASR expansion by 32% at 28 days.

Alkali silica gel expansions decreased significantly with the addition of SBR powder. Samples with a higher percentage of SBR powder expanded in a limiting range of 0.10% at 14 days and 0.2% at 28 days after casting. The alkali-silica reactivity (expansion) of mortar bars was gradually reduced to an acceptable limit after adding SBR powder; SBR powder positively affects concrete’s durability. The reason for controlled alkali-silica reactions might be the improved microstructure of SBR-powder-modified concrete and decreased permeability, which means SBR modification does not allow much interaction of reactive silica of aggregate with free alkali in cement. SBR powder on hydration blocks nearby alkali from coming in contact with the reactive aggregate. Additionally, SBR may block moisture ingress to the interfacial transition zone (ITZ); water presence is necessary to form a gel and its expansion in ITZ. The ASR expansion of the bar is reduced by 57% at 10% SBR modification. Usually, very expensive lithium salts are used to control ASR. However, ultrafine SBR provides a more sustainable and cost-effective solution against the expansion and cracking caused by ASR.

**Drying shrinkage:**

Drying shrinkage is the contraction of the hardened concrete or mortar mixture due to capillary water loss caused by evaporation. It is a major issue in hot and dry weather. The test was conducted at almost 33 °C (91.4 °F) and 57% relative humidity, so shrinkages were relatively higher than at 20 °C (68 °F). Table 7 and Figure 5 show the drying shrinkage results. In drying shrinkage test samples, cement hydration progresses through a considerably long dry curing period. The evaporation of water causes contraction/shrinkage, resulting in the cracking of mortar bar samples. Figure 5 shows the drying shrinkage results. The drying shrinkage is reduced by increasing the SBR powder percentage. Using SBR, both shrinkage and the number of cracks are reduced visibly.

SBR reduces the permeability and further loss of water by evaporation. The excellent water retention quality of SBR due to the polymer nature and the blocking of capillaries by producing SBR agglomerates (also verified by SEM study in the following section) reduces the evaporation process. Thus, a lesser amount of evaporation helps reduce the shrinkage of bars. Drying shrinkage is reduced by almost 33% and 53% at 5% and 10% cement replacement by SBR powder, respectively.

Additionally, the higher tensile capacity (indirectly related to better flexural strength) may also cause lesser drying shrinkage cracking and a lower number and size of cracks. In other words, SBR-modified bars might have better resistance against tensile stresses along with a better water-retention property, whereas in control samples, these tensile stresses increase due to shrinkage, leading to cracking, internal wrapping, and external deflection. The results obtained are similar to Moodi’s (2018) findings of SBR’s potential against shrinkage [44] and dimensional stability against drying shrinkage [22,45].

**Sulfate resistivity:**

Table 7 and Figure 5 show the expansion values of the samples due to sulfate attacks.

Sulfate ingression results in the cracking of the concrete due to ettringite formation in hardened concrete. External sulfate ions in the presence of monosulfates and tricalcium aluminate produce hard needles of ettringite. The hardened concrete is unable to accommodate this expansion of ettringite needles and is cracked. SBR powder reduces the permeability by filling the pores (and reduces sulfate ion ingression in concrete), resulting in remarkably reduced sulfate attacks. Hence, the ettringite formation in hardened concrete due to external sulfate ingression is reduced. Sulfate action-induced expansion is reduced by 33% and 73% using 5% and 10% cement replacement by SBR powder. A similar study found that SBR latex provides excellent resistance against chloride ingress [40].

**Water absorption:**

The water absorption test (shown in Table 8) shows a decrease in values with an increase in the percentage of SBR powder. The results are in accordance with the author’s parallel study. The mechanism behind it is the improved pore structure and water performance of polymer-modified mortars. The SEM study also verified these results of very low permeability at higher SBR% in the following sections.

**Interrelation between Permeability and Durability Issues:**

Microstructure and pore characteristics due to additives (e.g., polymers) in concrete greatly influence the mechanical properties, permeability, and durability [46]. Due to external ions and water ingression (permeability), concretes and mortars may face different ubiquitous durability issues such as sulfate attacks, alkali-silica reactions, and corrosion due to their intrinsic permeability. It may result in further cracking, loss of strength, and even the failure of structures. Likewise, high temperatures may also cause higher drying shrinkage and cracking, which is deleterious for concrete health [47].

**Figure 7:**
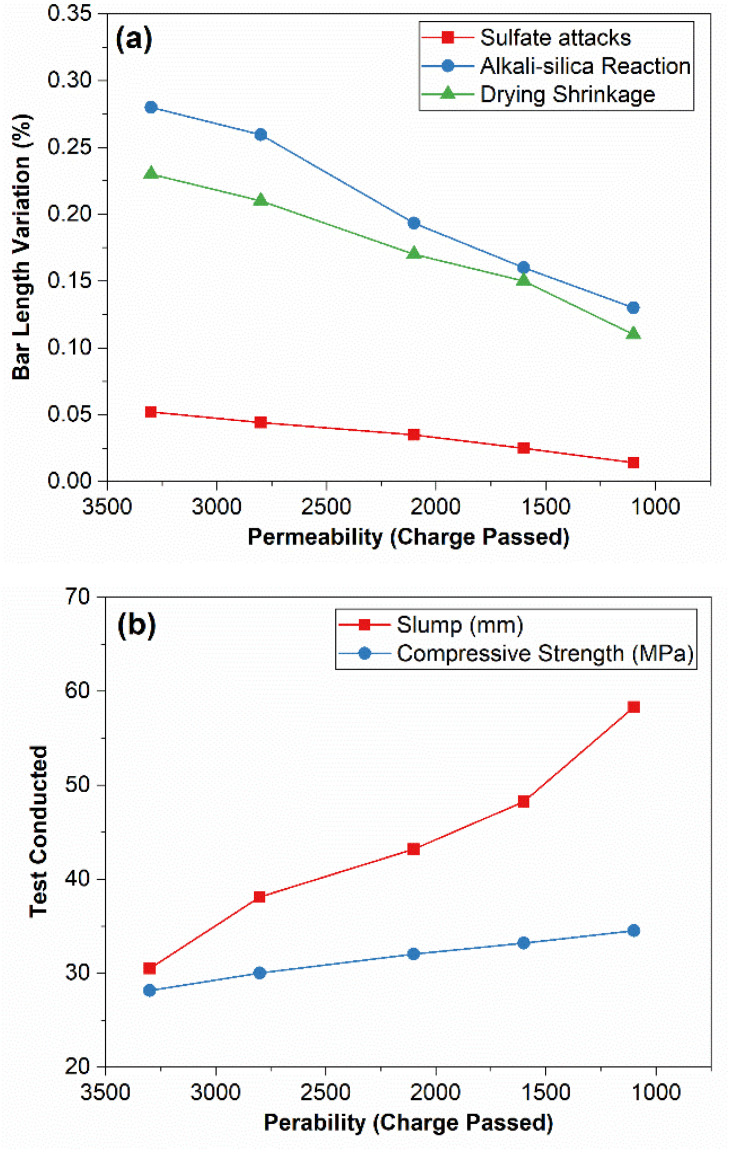
Interrelation between Chloride Permeability and (**a**) Durability Issues (**b**) Slump and Compressive Strength.

The permeability is directly or indirectly related to various properties of concrete, especially durability issues. Figure 7 presents the graph between charge passed in RCPT (representing chloride ion permeability) and durability issues of plain and SBR-modified concrete: drying shrinkage, expansion in bars due to sulfate attack, and bar expansion caused by the alkali-silica reaction. As permeability is a governing factor for durability issues, other factors are ignored as an assumption. The relations between permeability and concrete issues and attacks are found. The slump value and compressive strengths are also plotted against RCPT values. Following direct relations, Equations (1)–(3) are obtained between RCPT charge values and other properties. ASR (accelerated test) at 14 days, drying shrinkage at 28 days, and sulfate attack expansion at 15 weeks (time as recommended by ASTM standard tests) of SBR-modified concrete have the following relation with permeability.

Following relations are found:

(1) $$+{\Delta L}_{ASR}=4\times 10-5\;Q+0.0873\;$$

(2) $$-{\Delta L}_{DS}=3\times 10-5\;Q+0.0868$$

(3) $$+{\Delta L}_{SA}=1\times 10-5\;Q+0.0062$$

where ΔL is the change in length (%), −ve ΔL means shrinkage, while +ve ΔL means expansion. ΔL is recorded at 14 days for ASR accelerated test, 28 days for drying shrinkage test, as mentioned by following relevant ASTM standards, and at 15 weeks for normal sulfate reactions. Q is the charge passed at 360 min in RCPT experimentation. The above relations explain that durability issues such as alkali-silica reactivity, expansion caused by sulfate attacks, and drying shrinkage are aggravated with increased permeability. So, by controlling permeability, these perilous attacks on concrete can be reduced, and the lifespan of concrete can be efficiently increased. Thus, SBR powder is the key to controlling durability issues by reducing the permeability (RCPT values).

The following relations are obtained for the SBR-modified concrete.

(4) S =−7×10−4 Q+6.29

(5) C=−0.002Q +36.29

where S is slump (mm), C is compressive strength (MPa), and Q is charged passed at 360 min in RCPT tests. Increased slump and higher compressive strength in SBR-modified concrete are attributed to decreased permeability and porosity. A higher slump due to SBR polymer powder (not water) causes a reduction in the permeability because water is not evaporated easily, and interconnected pores are not produced. Pores are first filled with SBR powder and then with SBR-hydrated agglomerates. Fewer of pores with smaller sizes are formed. The water restrained in concrete by SBR powder might help in the internal curing and might contribute to the hydration process and strength gain. The lower permeability and overall lower porosity (observed in SEM image) result in higher compressive strengths.

### 3.4. Scanning Electron Microscopic (SEM) Studies

**Figure 8:**
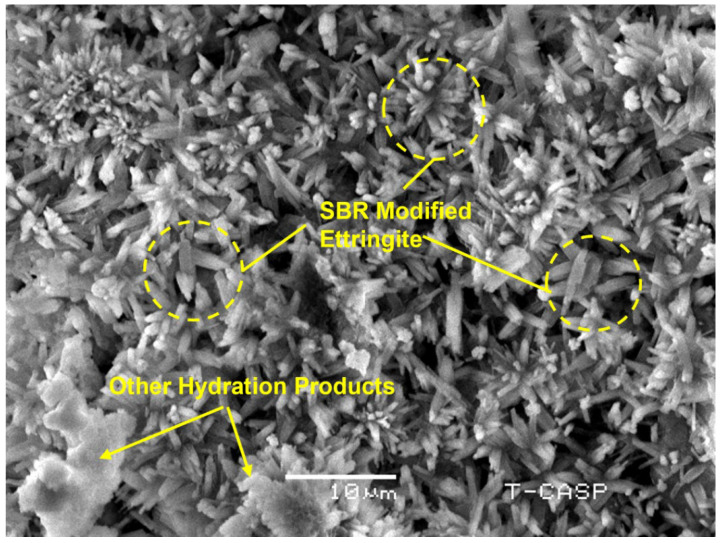
SEM Image of Mortar with 10% SBR. (Highly dense SBR wrapped ettringites needles).

Figure 8 reveals the SEM study of modified concrete with 10% SBR powder at a 10-micron scale. It reveals that the increase in strength and performance is a function of highly compact and dense concrete with very low permeability. The water absorption tests also corroborate the result. Furthermore, localized SBR powder particles (unlike liquid latex) did not stop the hydration of cement by not making a continuous membrane around cement particles. SBR ultrafine powder dispersed the cement particles and allowed them to hydrate fully. Localized hard agglomerate of SBR powder has either wrapped or bonded hydration products such as ettringite due to SBR’s adhesive nature. It also seems that SBR bonding/wrapping may not allow ettringite to change phase [29,48,49]. Sulfate reactions might be stopped due to the non-conversion of ettringites. Commonly, ettringites convert into monosulfates. Then, the conversion of monosulfates into ettringite again in the presence of sulfates and C_3_A is a major cause of sulfate reactions. Localized SBR powder agglomerates also blocked the capillaries, resulting in reduced permeability. The binding nature (adhesiveness) of SBR powder increased the strength. The non-formation of slippery membranes (unlike latex modification) prevented a reduction in strength that could be caused due to loss of friction. Meanwhile, smaller size hydrates that might be modified and strengthened by SBR powder are a possible cause of higher performance.

## 4. Conclusions

This study evaluates the effect of ultrafine styrene-butadiene-rubber (SBR) powder (up to 10%) with a particle size smaller than 85 microns as a partial cement replacement material on concrete issues and attacks in harsh environments. The results corroborate that the fresh and hardened properties of cementitious materials are enhanced significantly by incorporating SBR powder.

- The workability of concrete increases with the increase in the percentage of SBR powder. SBR (10%) increased slump value by 90%.
- The compressive strength of SBR-powder-modified concrete increases with increases in SBR percentage. SBR (10%) increased compressive strength by 23%. Moreover, flexural strengths of SBR-modified concrete increase slightly (9.4% for SBR10%) with an increase in the percentage of SBR.
- SBR powder reduces chloride permeability and provides protection against corrosion caused by chloride ingress. SBR (10%) decreased the charge passed and hence chloride permeability by one-third as compared to the control sample.
- SBR provides excellent volume stability by giving outstanding performance against dying shrinkage and expansions due to alkali-silica reactions and sulfate attacks. SBR (10%) reduced expansion in mortar bars due to sulfate attacks and ASR by 73% and 57%, respectively, and reduced shrinkage due to ASR by 52%.
- By increasing SBR powder from 5% to 10%, the durability issues are abated noticeably. The interrelation is developed for permeability and durability issues for SBR-modified concrete.
- Hence, it is inferred that concrete durability issues are substantially reduced by replacing cement with ultrafine SBR powder from 5 to 10% (especially at 10%). A highly sustainable and better-performance concrete is produced that is particularly suitable for harsh environments.

## Figures and Tables

**Table 1:** Properties of Cement and Aggregate.

**Sr.**	**Physical Properties of Cement**				**Obtained Value**			
1	Consistency				30			
2	Initial setting Time				110 min			
3	Final Setting Time				180 min			
4	Specific Gravity				3.13			
5	Fineness (Blain)				3078 cm^2^/gm			
6	Le-Chatlier Soundness				2.00 mm			
	**Chemical Properties of Cement**				**Obtained Value**			
1	Lime Saturated Factor				0.94			
2	Tri Calcium Aluminate				7.52%			
3	Insoluble Residue				1.28%			
4	Magnesia				1.79%			
5	Sulfuric Anhydride				2.76%			
6	Loss on Ignition				3.24%			
7	Chlorides				0.01%			
**XRF Analysis of Cement**								
**Material**	**CaO**	**MgO**	**SiO_2_**	**Al_2_O_3_**	**Fe_2_O_3_**	**L.O.I**	**K_2_O**	**Na_2_O**
Cement (%)	62.13	2.29	20.25	5.05	3.13	3.24	0.74	0.24
**Physical Properties of Aggregates**								
**Sr.**	**Material**		**Fineness Modulus**		**Specific Gravity**		**Water Absorption (Vol. %)**	
1	Coarse Aggregate		7		2.5		0.96	
2	Fine Aggregate		2.23		2.67		1.3	

**Table 2:** Chemical Properties of SBR Powder.

Property	Value	Unit	Method
Total Solids	99.00	%	ISO 1625
Ash Content at 600 °C	12.00	%	Internal Method
Particle Size	85.00	µm	Internal Method
MFFT (min. film-forming temperature for re-dispersed in 50% solid concentration)	8.00	°C	ISO 2115
Specific Gravity	0.50	g/cm^3^	ISO 8962

**Table 3:** Mix proportions of concrete samples.

Sample Name	Cement (kg)	SBR Powder (g)	Sand (kg)	Aggregate (kg)	Water (mL)
CS	4.25	0	7.22	10.62	1997
S3	4.12	127.5	7.22	10.62	1997
S5	4.04	212.5	7.22	10.62	1997
S7	3.95	297.5	7.22	10.62	1997
S10	3.83	425.0	7.22	10.62	1997

**Table 4:** Alkali-Silica Reaction Test: Mix Proportions and Expansions (ASTM C490 and ASTMC1260).

Sample Name	Cement (g)	SBR Powder (g)	Aggregate (g)	Water (mL)
CS	440	0	990	207
S3	426	14	990	207
S5	418	22	990	207
S7	409	31	990	207
S10	396	44	990	207

**Table 5:** Drying Shrinkage Test: Mix Proportions and Shrinkages at 33 °C (91.4°F) and 57% RH (ASTM C490 and ASTM C596).

Sample Name	Cement (g)	SBR Powder (g)	Sand (g)	Water (mL)
CS	300	0	600	207
S3	291	9	600	207
S5	285	15	600	207
S7	279	21	600	207
S10	270	30	600	207

**Table 6:** Sulfate Resistivity Test: Mix Proportions and Expansions (ASTM C490 and ASTM C1012).

Sample Name	Cement (g)	SBR Powder (g)	Sand (g)	Water (mL)
CS	1000	0	2750	485
S3	970	30	2.750	485
S5	950	50	2.750	485
S7	930	70	2.750	485
S10	900	100	2.750	485

**Table 7:** Variation in Length (%) of Mortar Bars Due to Expansions and shrinkage.

Sample Name	ASR Expansion (%)	ASR Decrease (%)	Drying Shrinkage (%)	DS Percent Decrease	Sulfate Attack Expansion (%)	Decrease in Expansion (%)
CS	0.28	-	0.218	-	0.52	-
S3	0.26	7.14	0.213	2.29	0.44	15.4
S5	0.19	32.14	0.147	32.5	0.35	32.7
S7	0.16	42.8	0.125	42.6	0.25	51.9
S10	0.12	57.14	0.103	52.7	0.14	73.1

**Table 8:** Permeability of concrete and mortars.

	Concrete (ASTM C1202)		Mortar	
Sample Name	Charge (Coulombs)	Decrease in Charge Passed (%)	Water Absorption (%)	Decrease (%)
CS	3300	-	7.9	-
S3	2899	12.15	4.1	49.3
S5	2100	36.36	3.5	55.7
S7	1600	51.51	3	62
S10	1100	66.67	1.5	81

## Data Availability

The data presented in this study are available upon request from the corresponding author.
